# Can in-hospital or post discharge caregiver involvement increase functional performance of older patients? A systematic review

**DOI:** 10.1186/s12877-020-01769-4

**Published:** 2020-09-22

**Authors:** Margaretha van Dijk, Jasmien Vreven, Mieke Deschodt, Geert Verheyden, Jos Tournoy, Johan Flamaing

**Affiliations:** 1 Department of Physical Medicine and Rehabilitation, UZ Leuven – University Hospitals Leuven, campus Pellenberg, Weligerveld 1, 3212 Pellenberg, Belgium; 2 Department of Public Health and Primary Care, Geriatrics and Gerontology, KU Leuven, Herestraat 49, 3000 Leuven, Belgium; 3 Nursing Science (INS), Department of Public Health, University of Basel, Bernoullistrasse 28, 4056 Basel, Switzerland; 4 Healthcare and Ethics, Faculty of Medicine and Life Sciences, UHasselt, Martelarenlaan 42, 3500 Hasselt, Belgium; 5 Department of Rehabilitation Sciences, KU Leuven, Tervuursevest 101, 3001 Leuven, Belgium; 6 Department Geriatric Medicine, UZ Leuven, Herestraat 49, 3000 Leuven, Belgium

**Keywords:** Caregiver involvement, Older adults, Functional performance, Hospitalization, Physiotherapy

## Abstract

**Background:**

Regaining pre-hospitalization activity levels is only achieved in 30–50% of older patients. Extra physiotherapy time has been proven to improve functional outcome and shorten length of stay, but is costly. Considering their key role in caring for older people, involving informal caregivers in rehabilitation might further improve functional performance.

**Aim:**

To determine if in-hospital or post discharge caregiver involvement can increase functional performance in older adults. The secondary aim was to determine if caregiver involvement can influence, quality of life of patient and caregiver, medical costs, readmission rate, discharge location, and mortality.

**Design:**

Systematic review with narrative synthesis.

**Methods:**

The electronic bibliographic databases MEDLINE, Embase, CINAHL, Cochrane and Web of Science were searched for (quasi) experimental and observational studies, with the following inclusion criteria; caregiver involvement regarding functional performance, mean age over 65 years, admitted to a hospital unit and subsequently discharged to their home setting. Risk of bias was assessed with the Rob 2 (randomized trials) and the ROBINS-1 tool (non-randomized studies).

**Results:**

Eight studies of an initial 4683 were included: four randomized controlled trials, one prospective cohort study, one non-randomized controlled trial, one subgroup analysis of an RCT and one prospective pre-post study. All but one study included patients with stroke. Three types of caregiver interventions could be distinguished: a care pathway (inclusion of caregivers in the process of care), education on stroke and teaching of bed-side handling-skills, and caregiver-mediated exercises. The one study evaluating the care pathway reported 24.9% more returns home in the intervention group. Studies evaluating the effect of education and bed-side handling-skills reported higher effect sizes for several outcomes with increasing session frequency. All studies with caregiver-mediated exercises showed beneficial effects on functional performance, immediately after the intervention and within 3 months follow-up.

**Conclusion:**

The findings of this review suggest that involvement of caregivers in the rehabilitation of older adults leads to better functional performance up to 3 months after initiation. However, evidence is low and mainly focusing on stroke.

## Background

Functional decline including reduced abilities of self-care are common consequences of hospitalization among older adults [1]. Up to 39% of patients 75 years or older admitted to geriatric, medical and surgical wards experience functional decline [2, 3]. The frequency of functional decline increases with age and is associated with the failure to recover to the basic activities of daily living (ADL) level during hospitalization [3]. Inactivity, immobility, suboptimal continence care, and poor nutrition during hospitalization have been suggested as the main reasons for functional decline [1, 4].

Physical therapy (PT) has proven to be effective in improving functional performance in older hospitalized patients [5]. A systematic review by Kosse et al. indicated that early physical therapy and rehabilitation can be safely executed and leads to better performance of ADL, shorter length of stay, and higher proportion of discharge home in hospitalized older adults [6]. Geriatric rehabilitation is best seen as a holistic treatment with a functional and multidisciplinary approach as well as long term health promotion [7–9]. Since increased physical activity is associated with arresting or reversing declines in functional performance, patients should be guided to seek opportunities and use available tools to achieve an active lifestyle [8, 9].

New hospital acquired ADL disability, is associated with the inability to regain pre-hospital functioning [10–12]. Only between 30 to 50% of the older patients regain pre-hospitalization activity levels and 25% of older adults treated in an inpatient rehabilitation facility are readmitted soon after discharge [13]. Limited time of PT, lack of patient activity outside therapy and detraining (deterioration of the acquired level of performance) when therapy has stopped are all reasons for functional decline, both during hospitalization and after discharge [14–17]. Extra PT time improves functional outcome and shortens length of stay, but consequently increases healthcare costs [18]. Even when PT meets the WHO activity guideline of 300 min of activity at moderate intensity per week, it still is insufficient to change sedentary behavior [19, 20]. At the same time, it is often difficult for hospitalized older patients to be active on their own, due to, among others, their dependency of healthcare workers (HCW) for transfers and walking. In addition, patients are discharged as soon as a basic level of functional performance is met, like getting in and out of bed [21]. Consequently, at the moment of discharge the patient’s functional performance level is lower than their pre-hospital level. This in turn, makes patients and caregivers cautious about taking up normal activities or even continuing exercises.

Informal caregivers play an important role in care of older people in Europe as no care system will ever be able to completely cover all long-term care needs by professional health care services [22]. A meta-analysis by Sörensen et al. shows that different interventions with caregivers are effective in lowering caregiver burden, depression, care receiver symptoms, and in improving subjective well-being, perceived caregiver satisfaction and ability/knowledge [23]. Although several studies evaluated the effect of caregiver involvement on functional performance in older adults, no systematic review on this subject has been undertaken.

The aims of this systematic review are:

1. To determine if in-hospital or post discharge caregiver involvement can increase functional performance in older adults.
2. To determine if caregiver involvement regarding functional performance can influence quality of life of patient and caregiver, need for professional help, readmission rate, discharge location, healthcare utilization and mortality.

## Methods

The review protocol was registered in PROSPERO (CRD42018091798) and the PRISMA reporting guidelines were used. (www.prisma-statement.org).

### Search strategy

Multiple search strategies were used for this review. The electronic bibliographic databases MEDLINE using PubMed, Embase, CINAHL, Cochrane and Web of Science were searched using a comprehensive search string on the fourth of December 2018 (Additional file 1). We limited our search to studies reporting in English, Dutch, German and French. There was no limitation with regard to the date of publication. In addition, the reference lists of relevant papers and related reviews were screened. Case reports, letters and editorials were manually excluded from the search results. On the sixth of April 2020 PubMed was searched again to identify potential new studies.

### Selection of relevant papers

All studies reporting on caregiver involvement regarding functional and physical performance activities in (recently) hospitalized older adults were of interest. Studies were included if they studied a sample of older adults aged 65 years or older (or with a mean age over 65 years old) admitted to a hospital unit and subsequently discharged to their home setting. Caregivers were defined as unpaid members of a person’s social network helping the patient with ADL. Studies using a randomized controlled design or a quasi-experimental design (including non-randomized controlled studies, before-and-after studies retrospective and prospective, and interrupted time series) with follow-up to twelve months were included.

Studies in nursing home residents and community dwelling older adults without a recent hospitalization, and reports on interventions with professional healthcare workers, were excluded. As standard of care can be expected to differ in light of international differences, no a-priori exclusion criteria were formulated in relation to the comparison group.

Two reviewers (JV, MV) screened the search results independently, using Endnote. First, titles and abstracts were screened, using the predetermined in- and exclusion criteria. If the titles and abstracts did not provide enough information the full text was read and screened independently by two reviewers to determine eligibility. In case of disagreement of study eligibility, the two reviewers discussed their findings and a third reviewer (MD) was consulted to reach an agreement.

### Data extraction and synthesis

One reviewer (JV) extracted data from the included studies and a second reviewer (MV) checked the completeness and correctness of the extracted data. The following data were extracted: first author, year of publication, country and setting, study design, study population, detailed description of the intervention (i.e. education, exercises, number of hours and/or sessions), outcome measures and definitions, time measurements, and follow-up after the end of intervention (Table 1). The results of the individual studies were tabulated and grouped according to four different outcomes (Tables 2 and 3 and Additional files 3 and 4). Results are summarized by means of a narrative synthesis.

**Table 1:** Characteristics

Author Year Country	Design (D) Setting (S) Sample size (SZ)	Target patient population	Intervention (I) Duration (D) Frequency (F)	Outcomes (O) Measure point (MP)
Everink et al. 2018 The Netherlands	D: Prospective cohort study S: Hospital, geriatric rehabilitation facility, community SZ: 149 patients 54 caregivers	Geriatric patients (> 65 years and complex health problems)Admitted to a geriatric rehabilitation facilityCommunity-dwelling prior to hospital admission	**I: Integrated Care Pathway**: Process of care during the trajectory of hospital admission, discharge to geriatric rehabilitation and discharge back to community. Patients and their caregiver are actively involved in the triage decision. D: Period of hospitalization (acute and rehabilitation) until discharge. F: Not stated	O: Patients:Basic functional performance (KI, FAI)Psychological well-being (CSAL)Caregivers:Psychological well-being (SRCB, CSAL) Others (Discharge location) MP: Admission geriatric rehabilitation, 3, 6 and 9 months
Forster et al. 2013 UK	D: Multicentre cluster RCT S: 36 stroke units in four geographical regions. SZ: 928 patients 928 caregivers	Patient with stroke Medically stable Likely to return home	**I: London Stroke Carers Training Course**:Assessment of competencies in knowledge or skills essential for day-to-day management of disabled survivors of stroke (14 components). Intervention manual and caregiver training record. D: Period of hospitalization F: Not stated	O: Patients:Basic functional performance (NEADL, BI) Psychological well-being (HADS, EQ-5D) Caregivers: Psychological well-being (CBS, HADS, EQ-5D)Others (initial stroke admission cost) MP: Measured at: Baseline, 6 and 12 months
Galvin et al. 2011 Ireland	D: RCT S: 6 acute hospitals SZ: 40 patients 40 caregivers	Patient with strokeNo cognitive impairment Participate in a physiotherapy program	**I: Family-Mediated Exercise Intervention**:Training the family member/friend with the skills necessary to carry out the exercise-training programme with the patient.Lower limb exercises designed to patient’s ability. Emphasis on achieving stability, gait velocity and strength D: 8 weeks F: Training the caregiver on a weekly basisExercises patient-caregiver 35 min daily	O: Patients:Basic functional performance (NEADL, BI, RNLI) Extended functional performance (LL-FMA, MAS, BBS, 6MWT)Caregivers:Psychological well-being (CSI) MP: Measured at: Baseline, 8 weeks and 3 months
Gräsel et al. 2005 Germany	D: Non-randomized controlled trialS: 2 study wards of a rehabilitation clinic SZ: 71 patients 71 caregivers	Patient with strokeFunctional deficit Required treatment in rehabilitation clinic	**I: Intensified Transition Concept**:Psycho-educational seminar for family carers.Individual training course on bedside skills.Therapeutic weekend care, accompanied and monitored by an outpatient care service.Telephone counselling to assess the home situation D: Duration of hospitalization plus 3 months after discharge F: 1-h psycho-educational seminar3 times 45–60 min individual training course1 therapeutic weekend1 telephone counselling after 3 months	O: Patients:Basic functional performance (BI, FIM)Extended functional performance (TUG, ASS, FAT) Caregivers:Psychological well-being (BSFC, ZDS, GSL)Others (discharge readmission) MP: Measured at: After intervention, 4 weeks and 6 months
Harris et al. 2010 Canada	D: Subgroup analysis of a RCT S: Multi-site SZ: 50 patients 50 caregivers	Patient with strokeActive scapular elevationFM scale 10–57	**I: Graded Repetitive Arm Supplementary Program with Caregiver Support**:A self-administered upper-limb exercise program, using an exercise booklet and a kit tailored to motor impairment level.Exercises included range of motion, strengthening, and fine motor and goal-directed activities. D: 4 weeks F: Explanation of the program 1 hExercises 60 min a day, 6 days per weekCaregiver involvement > 2 times/week	O: Patients:Extended functional performance (CAAI, MAL, GS) MP: Measured at: Baseline and 4 weeks
Hebel et al. 2014 Poland	D: Prospective pre-post study S: Hospital SZ: 243 patients 243 caregivers	Patient with stroke	**I: Voluntary Health Education Program for Carers**:Education on stroke and secondary prevention, proper patient positioning in bed and position changing techniques. D: During hospitalization F: One two-hour meeting	O: Patients:Basic functional performance (NEADL, BI, MRS) MP: Measured at: After intervention, 3 and 12 months
Kalra et al. Patel et al. 2004UK	D: RCTblock randomisation S: Hospital, home setting SZ: 300 patients 300 caregivers	Patient with strokeIndependent in ADL before strokeMedically stableExpected to return home	**I: Training Caregivers of Stroke Patients**:Instructions on common stroke related problems, hands-on training in lifting and handling techniques, facilitation of mobility and transfers, tailored to the needs of individual patients. D: During hospitalizationF: 3–5 session of 30–45 min1 follow through session at home	O: Patients:Basic functional performance (BI, MRS, FAI) Psychological well-being (HADS, EQ VAS) Caregivers:Psychological well-being (CBS, HADS, EQ VAS)Others (length of stay, cost, readmission, mortality, discharge destination) MP: Measured at: Baseline, 1, 3, 6 and 12 months
van den Berg et al. 2016 Australia	D: RCTS: Three hospitals and home setting SZ: 63 patients 63 caregivers	Patient with stroke Early rehabilitationMobility problemsNo cognitive problems No depression	**I: Caregiver-Mediated Exercises**:A customized exercise app (37 exercises) on a i-pad was provided to the patient and carer.Tele-rehabilitation services after discharge and weekly home visits. D: 8 weeks (hospital and home) F: ≥5 times per week 30 minweekly evaluation session with PT	O: Patients:Basic functional performance (NEADL, BI, MRS) Extended functional performance (SISmob, RMI, LL-FMA, MI, TUG, BBS) Psychological well-being (HADS)Caregivers:Psychological well-being (CSI, HADS) Others (Length of stay, Hospital readmission) MP: Measured at: Baseline, 8 weeks and 12 weeks

*6MWT* Six minute walk test, *ASS* Ashworth Spasticity Scale, *BBS* Berg Balance Scale, *BI* Barthel Index, *BSFC* Burden Scale for Family Carers, *CAAI* Chedoke Arm and Hand Activity Inventory, *CBS* Caregiver Burden Scale, *CSI* Caregiver Strain Index, *CSAL* Cantril’s Self Anchoring Ladder, *CSRI* Client Service Receipt Inventory, *EQ VAS* European Quality of Life Visual Analog Scale, *EQ-5D* European Quality of Life 5 Descriptive, *FAI* Frenchay Activities Index, *FAT* Frenchay Arm Test, *FIM* Functional Independence Measure, *GS* Grip strength, *GSL* Giessen Symptom List, *HADS* Hospital Anxiety and Depression Scale, *KI* Katz index, *MAL* Motor Activity Log, *MAS* Motor Assessment Scale, *MI* Motricity index, *MRS* Modified Rankin Scale, *NEADL* Nottingham Extended ADL, *RMI* Rivermead mobility index, *RNLI* Reintegration to Normal Living Index, *SISmob* Stroke Impact Scale mobility, *SRCB* Self Rated Caregiver Burden, *TUG* Timed up and Go, *ZDS* Zerssen Depression Scale

**Table 2:** Basic functional performance

Study Measure points		Basic ADL			Extended ADL			Others		
Everink et al 2018		**KI**, mean (SD)						**FAI**, mean (SD)		
T0= admission geriatric rehabilitation		T1	T2					T1	T2	
T1= 3 months	IG	4.6 (2.4)	4.4 (2.9)					31.1 (9.4)	31.0 (9.4)	
T2= 9 months	CG	5.7 (2.8)	5.0 (3.0)					27.4 (9.7)	29.4 (11.2)	
		*p* = 0.360	*p* = 0.862					***p*** **= 0.014**	*p* = 0.288	
Forster et al 2013					**NEADL,** mean (SE)					
T0= baseline					T1					
T1= 6 months	IG				27.4 (1.00)					
T2= 12 months	CG				27.6 (0.99)					
					*p* = 0.866					
Galvin et al 2011		**BI**, mean change (SD)			**NEADL**, mean change (SD)			**RNLI**, mean change (SD)		
T0= baseline		T1 - T0	T2 - T1		T2 – T1			T2 – T1		
T1= 8 weeks	IG	32.3 (24)	3.8 (8.3)		7.6 (8.3)			4.7 (4.3)		
T2= 3 months	CG	16.3 (14.2)	1.5 (11.6)		3.6 (7.8)			0.4 (2.9)		
		***p*** **= 0.04**	*p* = 0.36		***p*** **= 0.02**			***p*** **= 0.00**		
Gräsel et al 2005		**BI**, mean change (SD)								
		T2 – T0								
T0= after intervention	IG	11.4 (14.1)								
T2= 6 months	CG	11.2 (16.4)								
		*p* = 0.968								
		**FIM**, mean change (SD)								
		T2 – T0								
	IG	2.5 (12.9)								
	CG	7.4 (12.2)								
		*p* = 0.129								
Hebel et al 2014		**BI**, median			**NEADL**, median			**MRS**, median		
T0= after intervention		T0	T1	T2	T1	T2		T0	T1	T2
T1= 3 months	IG	60	75	90	7	14		3	2	2
T2= 12 months	CG	72.5	85	90	13	14.5		2	2	2
		***p*** **= 0.02**	*p* = 0.07	*p* = 0.65	***p*** **= 0.004**	*p* = 0.27		*p* = 0.11	*p* = 0.18	*p* = 0.53
Kalra/Patel et al 2004		**BI**, BI >18						**FAI**, median (IQR)		
T0= baseline		T2	T4					T0	T4	
T2= 12 weeks	IG	51.3%	61.5%					25 (20 - 29)	15 (9 - 23)	
T4= 52 weeks	CG	34.8%	50.3%					24 (21 - 29)	16 (8 - 22)	
		***p*** **= 0.007**	*p* = 0.074					*p* = not stated	*p*= not stated	
van den Berg et al 2016		**BI**, mean (95% CI)			**NEADL**, mean (95% CI)					
T0= baseline		T1	T2		T1	T2				
T1= 8 weeks	IG	89.3 (81.6 - 97)	89.4 (81.7 - 97.1)		14.3 (12.1-16.4)	15.9 (13.8-18.1)				
T2= 12 weeks	CG	84.9 (78.7 - 91)	88.7 (82.4 - 94.9)		10.7 (9 - 12.4)	12.9 (11.1-14.6)				
		*p* = 0.3811	*p* = 0.8894		***p*** **= 0.0118**	***p*= 0.0319**				

*IG* Intervention Group, *CG* Control Group, *KI* Katz Index, *BI* Barthel Index, *FIM* Functional Independence Measure, *NEADL* Nottingham Extended Activities of Daily Living, *FAI* Frenchay Activity Index, *RNLI* Reintegration to Normal Living Index, *MRS* Modified Rankin Scale

**Table 3:** Extended functional performance

Study Measure points		Lower limb Walking			Upper limb		Others		
Galvin et al. 2011		**LL-FMA,** mean change (SD)			**MAS**, mean change (SD)		**BBS**, mean change (SD)		
T0= baseline		T1- To	T2-T1		T1-T0	T2-T1	T1-T0	T2-T1	
T1= 8 weeks	IG	9.5 (9.9)	1.6 (2.4)		11.9 (7.8)	37.9 (9.7)	22.8 (18.1)	0.9 (2.5)	
T2= 3 months	CG	1.75 (6.3)	1.3 (5.2)		4.75 (6.2)	35.2 (10.8)	9 (9)	1.8 (8.5)	
		***p*** **= 0.01**	*p* = 0.12		***p*** **= 0.00**	*p* = 0.59	***p*** **= 0.02**	*p* = 0.7	
		**6MWT**, mean change (SD)							
		T1-T0	T2-T1						
	IG	164.1 (128.7)	39.8 (55.4)						
	CG	47.2 (50.6	-3.5 (32.7)						
		***p*** **= 0.00**	***p*** **= 0.01**						
Gräsel et al. 2005		**TUG**, number possible (%)			**FAT**, mean change (SD)		**ASS**, mean change (SD)		
T0= after intervention		T0	T2		T2-T0		T2-T0		
T1=4 weeks	IG	26 (79%)	31 (94%)		0.3 (1.5)		0.3 (1.0)		
T2= 6 months	CG	23 (79%)	22 (76%)		0.2 (0.8)		0.0 (1.0)		
		*p* = 0.960	*p* = 0.044		*p* = 0.679		*p* = 0.27		
Harris et al. 2010					**MAL**, mean change (SD)		**GS**, mean change (SD)		
T0= baseline					T1-T0		T1-T0		
T1= 4 weeks	IG				2.1 (0.72)		5.8 (3.1)		
	CG				1.0 (0.78)		3.4 (2.4)		
					***p*** **= 0.024**		***p*** **= 0.034**		
					**CAAI**, mean change (SD)				
					T1-T0				
	IG				20.6 (6.1)				
	CG				15.0 (7.3)				
					***p*** **= 0.021**				
van den Berg et al. 2016		**LL-FMA**, mean (95% CI)					**BBS**, mean (95% CI)		
T0= baseline		T0	T1	T2			T0	T1	T2
T1= 8 weeks	IG	19.4 (16.3 - 22.6)	26.1 (23 - 29.2)	26.1 (22.9 - 29.2)			31.8 (26 - 37.6)	50.2 (44.3 - 56)	49.8 (43.9 - 55.6)
T2= 12 weeks	CG	17.2 (14.8 - 19.7)	22.4 (19.3 - 24.9)	27.6 (24.9 - 30.3)			26.7 (22.1 - 31.3)	44.3 (39.7 - 49)	46.3 (41.6 - 51)
		*p* = 0.2654	*p* = 0.0721	*p* = 0.4577			*p* = 0.1752	*p* = 0.1275	*p* = 0.3681
		**TUG**, mean (95% CI)					**SISmob**, mean (95% CI)		
		T0	T1	T2			T0	T1	T2
	IG	34.2 (28.6 - 39.8)	18.2 (12.6 - 23.8)	17.5 (11.9 - 23.2)			44.8 (36.8 - 52.8)	82.3 (74.3 - 90.3)	82.5 (74.5 - 90.4)
	CG	44.2 (39.7 - 48.7)	17.5 (12.9 - 22.1)	14.1 (9.2 - 19)			43.1 (36.8 - 49.4)	72.5 (66 - 78.9)	74.7 (68.2 - 81.2)
		***p*** **= 0.0075**	*p* = 0.8503	*p* = 0.3704			*p* = 0.7342	*p* = 0.06	*p* = 0.1382
							**RMI**, mean (95% CI)		
							T0	T1	T2
	IG						7.7 (6.3 - 9.1)	12.6 (11.2 - 14)	12.6 (11.2 - 14)
	CG						6.8 (5.6 - 7.9)	11.6 (10.5 - 12.8)	12 (10.8 - 13.1)
							*p* = 0.2937	*p* = 0.284	*p* = 0.5245
							**MI**, mean (95% CI)		
							T0	T1	T2
	IG						66.5 (60.1 - 72.9)	78.5 (72.1 - 84.9)	78.9 (72.5 - 85.3)
	CG						62.4 (57.4 - 67.5)	74.2 (69.1 - 79.3)	83.5 (78.2 - 88.8)
							*p* = 0.3291	*p* = 0.3058	*p* = 0.2814

*IG* Intervention Group, *CG* Control Group, *LL-FMA* Lower Limb Fugl Meyer Assessment, *TUG* Timed Up and Go test, *MAS* Motor Assessment Scale, *FAT* Frenchay Arm Test, *MAL* Motor activity Log, *CAAI* Chedoke Arm and Hand Activity Inventory, *BBS* Berg Balance Scale, *6MWT* 6 minute walk test, *ASS* Ashworth Spasticity Scale, *GS* grip strength, *SISmob* Stroke Impact Scale mobility part, *RMI* Rivermead Mobility Index, *MI* Motricity Index

Risk of bias of randomized controlled trials (RCTs) and cluster RCTs was evaluated by one reviewer (MD) at the study level with the revised Cochrane risk of bias tool (Rob 2) which grades the risk of selection, performance, attrition, detection and reporting bias, while risk of bias of the non-randomized studies was evaluated with the ROBINS-I tool [24, 25].

## Results

### Article selection

**Fig. 1:**
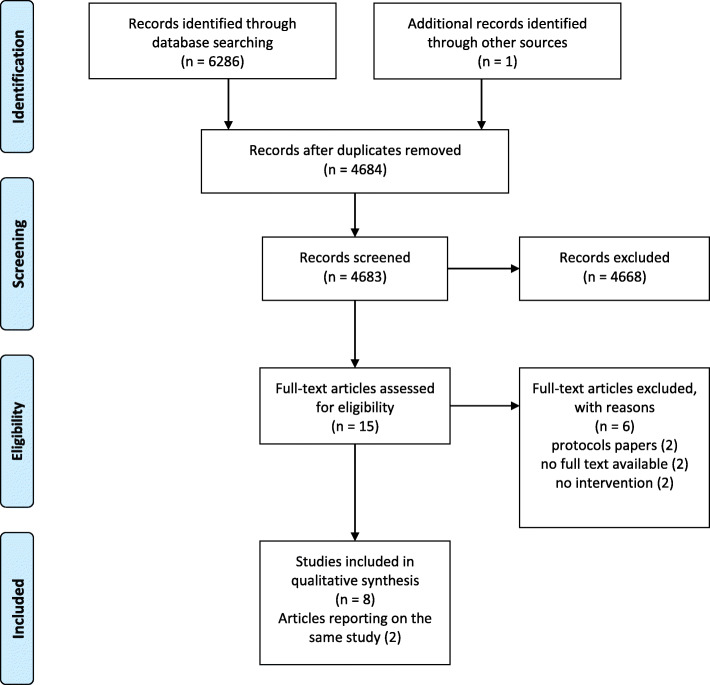
PRISMA flowchart

A total of 4683 studies were screened for eligibility (Fig. 1). After excluding 4669 articles based on title and abstract, 15 full-texts were read to determine eligibility. A total of nine articles reporting on eight studies were analyzed [26–34]. Two articles reported on the same study and subjects and they will be referred to as one study [32, 33]. Additionally, two protocol papers of included studies were available, which we used for data on characteristics and risk of bias [35, 36]. Another 144 studies were screened for eligibility after the search on the sixth of April 2020, but none was withheld.

### Risk of bias

The four included RCTs did not show a high risk of any form of bias on any domain, but there were concerns regarding performance and detection bias [27, 28, 32–34]. The non-randomized studies all showed a high risk of bias due to confounding [26, 29–31]. Two of the non-randomized studies also scored high for selection bias [30, 31] and two scored high on bias due to missing data [26, 31]. Additional file 2 shows all the results of risk of bias assessment.

### Characteristics of the included studies

We included four RCTs [27, 28, 32–34], one prospective cohort study [26], one non-randomized controlled trial [29], one subgroup analysis of an RCT [30] and one prospective pre-post study [31]. Six of the studies were performed in Europe [26–29, 31–33], one in Canada [30] and one in Australia [34]. Seven studies included only patients with stroke [27–34] and one included geriatric patients with complex health problems [26]. Sample sizes varied from 40 to 928 patient-caregiver dyads. One study had no follow-up (last measurement was at the end of the intervention) [30] and three studies had 12-month follow-up [27, 31–33]. In two studies the first outcome measure point, which was called baseline, was at discharge, after the intervention [29, 31]. The study of Forster et al. evaluated the same intervention as Kalra & Patel et al., but in another setting and other study sample [27, 32, 33]. More information regarding the included studies can be found in Table 1.

### Reported outcome measures

All studies measured functional performance, which in turn can be subdivided into basic functional performance (ADL) and extended functional performance. Six studies also used a variety of other outcomes (caregiver burden, quality of life, patient and/or caregiver depression, length of stay and initial hospitalization costs), which can be grouped into psychological well-being and others.

#### Basic functional performance

Basic functional performance was most often measured by the Barthel Index (BI) [28, 29, 31–34] and the Nottingham Extended ADL [27, 28, 31, 34]. Other outcome measures used were the Frenchay Activities Index (FAI) [26, 32, 33], the Katz index (KI) [26], the Reintegration to Normal Living Index (RNLI) [28] and the Modified Rankin Scale (MRS) [31]. An overview can be found in Table 2.

#### Extended functional performance

Extended functional performance outcome measures were quite diverse and nearly every study used different ones. Lower limb function was measured with the Lower Limb of Fugl Meyer Assessment (LL-FMA) [28, 34], gait speed with the Timed up and Go (TUG) [29, 34] and the six-minute walk test (6MWT) [28] and balance with the Berg Balance Scale (BBS) [28, 34]. Upper limb function was measured with the Chedoke Arm and Hand Activity Inventory (CAAI) [30], the grip strength (GS) [30] and the Frenchay Arm Test (FAT) [29]. General mobility was measured with the Motor Activity Log (MAL) [30], the Motor Assessment Scale (MAS) [28], Stroke Impact Scale mobility (SISmob) [34], Rivermead mobility index (RMI) [34] and the Motricity index (MI) [34]. Furthermore, the Ashworth Spasticity Scale (ASS) [29] was used to measure spasticity. An overview can be found in Table 3.

#### Psychological well-being

Caregiver burden was measured in six studies by means of the Caregiver Burden Scale (CBS) [27, 32, 33], the Caregiver Strain Index (CSI) [28, 34], the Self Rated caregiver burden (SRCB) [26], or the Burden Scale for Family Carers (BSFC) [29]. Depression was measured with the Hospital Anxiety and Depression Scale (HADS) in both caregiver and patient [32–34], or with the Zerssen Depression Scale (ZDS) [29]. Furthermore, quality of life was measured with the Euro Quality of Life (EQoL) [27] and the European Quality of Life Visual Analog Scale (EQ VAS) [32, 33]. Other measurements used for psychological well-being were the Giessen Symptom List (GSL) [29] and the Cantril’s Self Anchoring Ladder (CSAL) [26]. An overview can be found in Additional file 3.

#### Other outcomes

Healthcare utilization was evaluated as either length of stay [32–34], stroke hospitalization costs [27, 32, 33], total annual health and social care costs [32, 33]. Other outcomes were readmission rate [27, 29, 32–34], mortality [27, 32, 33] and discharge location [26, 27, 32, 33]. An overview can be found in Additional file 4.

### Interventions

The caregiver interventions ranged from including the caregiver in the decision-making process of discharge planning, to letting the caregiver deliver exercises. All interventions were initiated in the hospital, but their duration ranged from 2 hours to 8 weeks and in three studies the intervention continued after discharge. Studies were grouped into three different types of caregiver intervention.

#### Care pathway

One study was categorized in the care pathway group and focused on the process of care. Patients and their caregiver are actively involved in the triage decisions (discharge to a geriatric rehabilitation facility and discharge back to the community) and in the establishment of their care and treatment plan [26].

#### Education and bed-side handling skills

Four studies evaluated caregiver education and teaching bed-side handling skills, such as education regarding stroke (consequences and prevention) and teaching skills for day-to-day management of stroke survivors like positioning in bed and transfers [27, 29, 31–33]. The duration of these in-hospital interventions was a two-hour meeting [31], 3–5 sessions of 30–45 min [32, 33], or 4 times 45–60 min [29], Gräsel et al. conducted additional telephone counseling up to 3 months after discharge [29].

#### Caregiver mediated exercises

Three studies reported on caregiver mediated exercises, were caregivers assist the patient with a self-administered exercise program supported with a booklet or an app [28, 30, 34]. Galvin et al. studied exercises to strengthen lower limb with the intention of improving balance and gait velocity [28]. Harris et al. focused on improving functionality of the upper limb [30]. Van den Berg et al. studied the exercises aiming to improve gait and gait-related mobility [34]. The duration of these interventions was 4 weeks 60 min a day 6 times per week [30], 8 weeks 35 min a day [28] or 8 weeks ≥5 times per week 30 min [34]. In two studies the intervention continued after discharge [28, 34].

### Impact of intervention types

#### Care pathway

Everink et al. reported significant improvements for basic functional performance (FAI) and psychological well-being (SRCB) at 3 months, but not at 6 months or 9 months. More intervention patients (83% versus 58%, *p* = 0.004) could be discharged home [26].

#### Education and bed-side handling skills

Kalra & Patel et al. reported significant improvements for basic functional performance at 12 weeks for the BI, but not for the FAI [32, 33]. However, Hebel et al. found significant differences at the end of the hospitalization (approximately 3 days after the intervention in favor of the control group (BI), but these differences faded at follow-up [31]. Gräsel et al. evaluated extended functional performance (TUG, ASS, FAT) but did not observe significant differences [29]. The RCT of Kalra & Patel et al. found a positive effect on psychological well-being for patients and caregivers on all outcome measures used (CBS, HADS, EQVAS) [32, 33], while the RCT of Forster et al. and the non-randomized controlled trial of Gräsel et al. did not (CBS, HADS, EQ-5D, BSFC, GSL, ZDS) [27, 29]. The utilization of healthcare resources was significantly reduced in the study of Kalra & Patel et al. (length of stay, initial admission costs and total annual health- and social care costs), but not in the study of Forster et al. (stroke hospitalization costs) [27, 32, 33]. In contrast, more readmissions occurred in the intervention group in the study by Gräsel at 4 weeks, but this difference was not found at 6 months [29].

#### Caregiver mediated exercises

Galvin et al. and van den Berg et al. evaluated basic functional performance and found significant improvements in favor of the intervention group, for different outcome measures (BI, NEADL, RNLI) and at different time points [28, 34]. Significant positive results were found on all outcome measures used for extended functional performance by Galvin et al. (LL-FMA, 6MWT, MAS, BBS) and Harris et al. (MAL, CAAI, grip strength), but not by van den Berg et al. (SIS, RMI, LL-FMA, MI, TUG, BBS) [28, 30, 34]. Psychological well-being was found to be positively influenced in the study of Galvin et al. (CSI). However, van den Berg only found improvement in one of three psychological outcomes (HADS) [28, 34]. Van den Berg et al. also looked at the use of health resources and found significant differences in favor of the intervention group for length of stay and readmission [34].

## Discussion

This review summarizes the evidence of in-hospital and post discharge caregiver involvement in increasing functional performance in older adults. Furthermore, we looked at the influence on psychological well-being, healthcare utilization, discharge destination and mortality. Based on the main caregiver intervention we distinguished three groups, a care pathway [26], education and bed-side handling skills [27, 29, 32, 33], and caregiver mediated exercises [28, 30, 34]. The influence of caregiver involvement on functional performance, as well as on the other outcomes varied widely between the eight included studies. Positive results were mainly evident at the end of the intervention and in the following 3 months.

### Care pathway

The care pathway showed improvements on functional performance of the patients and lowering of the caregiver burden at 3 months. The care pathway might cover the unmet needs of family caregivers that McCusker et al. identified, namely patient medical information, role clarity and support, and reassurance [37]. Good communication about care needs and triage with the caregiver and the patient during hospitalization might make the caregivers more apt to give the support needed. Consequently, this could have positively influenced the number of patients that returned home.

### Education and teaching of bed-side handling-skills

Education on stroke and teaching of bed-side handling-skills showed a positive result on functional performance at 3 months in two of the four studies [32, 33]. Given that the other two studies only looked at functional performance at more than 6 months after the intervention, an earlier effect could have been missed [27, 29]. Secondly, best practices for stroke patient and family education, stress the importance of repetition in education [38]. Since training of the bed-side handling skills of the caregiver was only available during hospitalization, there might not have been enough learning opportunities. This was especially the case in de study of Hebel et al. were there was only one contact moment [31]. Also, the process evaluation of the Forster et al. study, showed that in general bed-side and transfer handling-skill were only practiced a few days before discharge and the caregiver mostly observed while therapists were actually performing the transfers [39].

The results on the effect on psychological well-being of education are conflicting. Where Kalra & Patel et al. found very positive results on all different measurements as well for patients as for caregivers, Forster et al. (using the same intervention) did not [27, 32, 33]. The more personal approach, intervention ownership and intervention fidelity of a small group people who deliver the intervention, compared to a large group of people in a multi-center study might explain these differences [39]. This is in line with Luker et al. who have shown that ownership of an intervention by staff members and patients is important to successfully implement a complex rehabilitation intervention in a clinical trial [40].

Concerning healthcare utilization, the shorter LOS and the lower stroke hospitalization costs in the intervention group of Kalra & Patel et al. study, could be linked to the better functional performance they found [32, 33]. This is in line with the studies on older adults in acute care settings where a discharge decision is based on patients functioning [41]. In the study of Forster et al. the discharge date was already known before the intervention was started and thus explains why no shorter LOS was found [39].

### Caregiver mediated exercises

Two of the three studies with caregiver mediated exercise interventions had positive results on all outcomes for functional performance. These results are in line with several reviews on caregiver-mediated exercises for patients with stroke, where extra therapy leads to better functional performance [18, 42, 43]. However, van den Berg only found positive results on two out of eight outcomes namely for the TUG and the NEADL. Looking at the baseline values, the intervention group (IG) of van den Berg et al. performed better compared to the IG of Galvin (an average of 69.6 compared to 56.3 for the BI respectively) [28, 34]. Patients with lower non-minimal baseline scores have greater potential for improvements [6].

The effect of caregiver mediated exercises on psychological well-being is unclear. These conflicting results might be explained by the many different reasons why a caregiver can experience burden. Besides patient’s functional performance there are other factors like anxiety and cognitive function, as well as caregivers’ characteristics like an individual’s ability to cope with stress, depression, anxiety and physical health [44]. The process evaluation of the study of Galvin et al. showed that the intervention was positively received, it gave structure in daily life and a sense of involvement in the recovery process [45], this explains the positive results they found in favor of the IG for caregiver burden (CSI) [28].

### Methodological considerations

Dividing the studies into groups according to their intervention type results in even smaller numbers of studies. However, we believe this distinction is important as the interventions do differ with regard to content and implementation. The quality of the studies ranges from low till moderate, resulting in low evidence. The different outcome measures used and variation in methodology refrained us from comparing the three different caregiver interventions with each other. Furthermore, seven of the eight studies included were on patients with stroke, and this limits the generalizability of our findings to the geriatric in-hospital and post discharge population.

Despite the small body of evidence regarding caregiver involvement in physiotherapy interventions, the results indicate a potential beneficial effect on several patient-related outcomes. Nevertheless, more high-quality studies are needed to confirm these findings given the very low grade of evidence, and more attention should be given to process evaluations investigating adherence to the intervention after discharge. Besides questionnaires, objective measurements should be used to evaluate basic functional performance.

## Conclusion

The findings of this review suggest that involvement of caregivers in the rehabilitation of older adults leads to better functional performance up to 3 months after initiation. Three different types of caregiver involvement showed positive results for functional performance up to 3 months after initiation, but evidence is low. Sessions on education and handling skills practice seem effective to improve functional performance and psychological well-being, and decrease length of stay. Caregiver-mediated exercises seem to be more effective when baseline functional performance is lower. However, further research is needed on the generalizability of and contributing factors to, these improvements as programs involving caregivers are so far heterogeneous, predominantly investigated post stroke and show a variable effect on outcomes.

## Supplementary information

**Additional file 1.**

**Additional file 2.**

**Additional file 3.**

**Additional file 4.**

## Data Availability

Not applicable.

## Abbreviations

6MWT: Six minutes’ walk test
ASS: Ashworth Spasticity Scale
BBS: Berg Balance Scale
BI: Barthel Index
BSFC: Burden Scale for Family Carers
CAAI: Chedoke Arm and Hand Activity Inventory
CBS: Caregiver Burden Scale
CG: Control Group
CME: Caregiver-Mediated Exercises
CP: Care Pathway
CSI: Caregiver Strain Index
CSAL: Cantril’s Self Anchoring Ladder
CSRI: Client Service Receipt Inventory
E&BHS: Education and Bed-site Handling Skills
EQ: VAS European Quality of Life Visual Analog Scale
EQ-5D: European Quality of Life 5 Descriptive
FAI: Frenchay Activities Index
FAT: Frenchay Arm Test
FIM: Functional Independence Measure
GS: Grip strength
GSL: Giessen Symptom List
HADS: Hospital Anxiety and Depression Scale
HCW: Healthcare worker
IG: Intervention group
KI: Katz index
LOS: Length of Stay
MAL: Motor Activity Log
MAS: Motor Assessment Scale
MI: Motricity index
MRS: Modified Rankin Scale
NEADL: Nottingham Extended ADL
PT: Physical therapist
RMI: Rivermead mobility index
RNLI: Reintegration to Normal Living Index
SISmob: Stroke Impact Scale mobility
SRCB: Self Rated Caregiver Burden
TUG: Timed up and Go
ZDS: Zerssen Depression Scale
