# Supplementary material for: Can in-hospital or post discharge caregiver involvement increase functional performance of older patients? A systematic review
Source: BMC Geriatr. 2020 Sep 22;20:362. doi: 10.1186/s12877-020-01769-4 (PMC7510152; doi:10.1186/s12877-020-01769-4)
Supplement: Supplementary file 1 — Additional file 1. [file 12877_2020_1769_MOESM1_ESM.docx]

**Appendix 1: search string**

**Pubmed**

(aged[tiab] OR elder*[tiab] OR old[tiab] OR older[tiab] OR senior[tiab] OR seniors[tiab] OR frail*[tiab] OR aged-80-and-over[tiab] OR geriatric*[tiab]) AND **(**"Caregivers"[Mesh] OR caregiver*[tiab] OR care-giver*[tiab] OR carer*[tiab] OR "Family"[Mesh] OR family[tiab] OR families[tiab] OR Spouse[tiab] OR spouses[tiab] OR relatives[tiab] OR relative[tiab] OR "Social Support"[Mesh:NoExp] OR social-support[tiab] OR "Volunteers"[Mesh:NoExp] OR volunteers[tiab] OR volunteer[tiab] OR voluntary[tiab]) AND **(**daily-living[tiab] OR activities-of-daily-living[tiab] OR ADL[tiab] OR daily-life-activity*[tiab] OR functional-performance[tiab] OR functional-training[tiab] OR functional-status[tiab] OR motor-performance[tiab] OR functionality[tiab] OR functional-state[tiab] OR functional-condition[tiab] OR functional-capacity[tiab] OR functional-capacities[tiab] OR physical-capacity[tiab] OR physical-capacities[tiab] OR functional-change*[tiab] OR recovery-of-function[tiab] OR self-care-activities[tiab] OR independent-living[tiab]) AND **(**"Hospitalization"[Mesh:NoExp] OR hospitalization[tiab] OR hospitalizations[tiab] OR hospitalisation[tiab] OR hospitalisations[tiab] OR hospitalized[tiab] OR “Patient discharge”[Mesh] OR patient-discharge[tiab] OR discharge[tiab] OR "Aftercare"[Mesh:NoExp] OR aftercare[tiab] OR follow-up[tiab] OR after-treatment[tiab] OR "Postoperative Period"[Mesh:NoExp] OR postoperative[tiab]).

**Embase**

aged:ti,ab,kw OR elder*:ti,ab,kw OR old:ti,ab,kw OR older:ti,ab,kw OR senior:ti,ab,kw OR seniors:ti,ab,kw OR frail*:ti,ab,kw OR geriatric*:ti,ab,kw OR ‘aged 80 and over’:ti,ab,kw AND 'caregiver'/exp OR caregiver*:ti,ab,kw OR ‘care giver*’:ti,ab,kw OR carer*:ti,ab,kw OR 'family'/exp OR family:ti,ab,kw OR families:ti,ab,kw OR 'spouse'/exp OR spouse:ti,ab,kw OR spouses:ti,ab,kw OR 'relative'/exp OR relatives:ti,ab,kw OR 'social support'/exp OR ‘social support’:ti,ab,kw OR 'volunteer'/exp OR volunteer:ti,ab,kw OR volunteers:ti,ab,kw OR voluntary:ti,ab,kw AND 'daily life activity'/exp OR ‘daily living’:ti,ab,kw OR ADL:ti,ab,kw OR ‘activities of daily living’:ti,ab,kw OR 'functional performance'/exp OR ‘motor performance’:ti,ab,kw OR ‘functionality’:ti,ab,kw OR ‘functional performance’:ti,ab,kw OR 'functional training'/exp OR ‘functional training’:ti,ab,kw OR 'functional status'/exp OR ‘functional status’:ti,ab,kw OR ‘functional capacity’:ti,ab,kw OR ‘functional capacities’:ti,ab,kw OR ‘functional-state’:ti,ab,kw OR ‘functional-condition’:ti,ab,kw OR ‘functional change*’:ti,ab,kw OR ‘physical capacity’:ti,ab,kw OR ‘physical capacities’:ti,ab,kw OR ‘self-care activities’:ti,ab,kw OR ‘recovery of function’:ti,ab,kw OR ‘independent-living’:ti,ab,kw AND 'hospitalization'/exp OR hospitalization:ti,ab,kw OR hospitalizations:ti,ab,kw OR hospitalisation;ti,ab,kw OR hospitalisations:ti,ab,kw OR hospitalized:ti,ab,kw OR discharge:ti,ab,kw OR ‘patient discharge’:ti,ab,kw OR 'aftercare'/exp OR aftercare:ti,ab,kw OR ‘follow up’:ti,ab,kw OR ‘after treatment’:ti,ab,kw OR 'postoperative period'/de OR postoperative:ti,ab,kw

**Web of science**

aged OR elder* OR old OR older OR geriatric* OR senior OR seniors OR frail* OR aged-80-and-over AND caregiver* OR care-giver* OR carer* OR family OR families OR Spouse OR spouses OR relatives OR relative OR social-support OR volunteers OR volunteer OR voluntary AND daily-living OR activities-of-daily-living OR ADL OR daily-life-activity OR functional-performance OR functional-training OR functional-status OR motor-performance OR functionality OR self-care-activities OR functional-state OR functional-condition OR functional-capacity OR functional-capacities OR physical-capacity OR physical-capacities OR functional-change* OR independent-living OR recovery-of-function AND hospitalization OR hospitalizations OR hospitalisation OR hospitalisations OR hospitalized OR discharge OR patient-discharge OR aftercare OR follow-up OR after-treatment OR postoperative OR postoperative-period

**Cochrane**

[mh “Geriatrics”] OR (aged OR elder* OR old OR older OR geriatric* OR senior OR seniors OR frail* OR aged 80 and over):ti,ab,kw AND [mh “Caregivers”] OR [mh “family”] OR [mh ^"Social Support"] OR [mh “volunteers”] OR (caregiver* OR care-giver* OR carer* OR family OR families OR Spouse OR spouses OR relatives OR relative OR social-support OR volunteers OR volunteer OR voluntary OR):ti,ab,kw AND [mh “recovery of function”] OR (daily-living OR activities-of-daily-living OR ADL OR daily-life-activity OR functional-performance OR motor-performance OR functionality OR self-care-activities OR functional-training OR functional-state OR functional-condition OR functional-status OR functional-capacity OR functional-capacities OR physical-capacity OR physical-capacities OR functional-change* OR independent-living OR recovery-of-function):ti,ab,kw AND [mh ^“hospitalization”] OR [mh ^”aftercare”] OR [mh ^“postoperative period”] OR (hospitalization OR hospitalizations OR hospitalisation OR hospitalisations OR hospitalized OR discharge OR aftercare OR follow-up OR after-treatment OR postoperative):ti,ab,kw

**CINAHL**

TI(aged OR elder* OR old OR older OR geriatric* OR senior OR seniors OR frail* OR aged 80 and over) OR AB(aged OR elder* OR old OR older OR geriatric* OR senior OR seniors OR frail* OR aged 80 and over) AND (MH "Caregivers") OR (MH "Family+") OR (MH “Spouses”) OR TI(caregiver* OR care-giver* OR carer* OR family OR families OR Spouse OR spouses OR relatives OR relative OR social-support OR volunteers OR volunteer OR voluntary) OR AB(caregiver* OR care-giver* OR carer* OR family OR families OR Spouse OR spouses OR relatives OR relative OR social-support OR volunteers OR volunteer OR voluntary) AND TI(daily-living OR activities-of-daily-living OR ADL OR daily-life-activity OR functional-performance OR functional-training OR motor-performance OR functionality OR self-care-activities OR functional-status OR functional-state OR functional-condition OR functional-capacity OR functional-capacities OR physical-capacity OR physical-capacities OR functional-change* OR independent-living OR recovery-of-function) OR AB(daily-living OR activities-of-daily-living OR ADL OR daily-life-activity OR motor-performance OR functionality OR self-care-activities OR functional-performance OR functional-training OR functional-status OR functional-state OR functional-condition OR functional-capacity OR functional-capacities OR physical-capacity OR physical-capacities OR functional-change* OR independent-living OR recovery-of-function) AND (MH “Hospitalization”) OR TI(hospitalization OR hospitalizations OR hospitalisation OR hospitalisations OR hospitalized OR discharge OR aftercare OR follow-up OR after-treatment OR postoperative) OR

AB(hospitalization OR hospitalizations OR hospitalisation OR hospitalisations OR hospitalized OR discharge OR aftercare OR follow-up OR after-treatment OR postoperative)
