# Supplementary material for: Can in-hospital or post discharge caregiver involvement increase functional performance of older patients? A systematic review
Source: BMC Geriatr. 2020 Sep 22;20:362. doi: 10.1186/s12877-020-01769-4 (PMC7510152; doi:10.1186/s12877-020-01769-4)
Supplement: Supplementary file 2 — Additional file 2. [file 12877_2020_1769_MOESM2_ESM.docx]

**Appendix 2. Risk of bias**

Risk of bias evaluation of randomised and cluster-randomised controlled trials using the Revised Cochrane risk-of-bias tool (RoB 2) [1]

|  | Random sequence generation (selection bias) | Allocation concealment (selection bias) | Deviations from intended protocol (performance bias) | Blinding of outcome assessment (detection bias) | Incomplete outcome data (attrition bias) | Selective reporting (reporting bias) |
| --- | --- | --- | --- | --- | --- | --- |
| Forster 2013 | Low | Low | Some concerns | Some concerns | Some concerns | Low |
| Galvin 2010 | Some concerns | Some concerns | Some concerns | Some concerns | Low | Low |
| Kalra/Patel 2004 | Low | Low | Some concerns | Some concerns | Low | Low |
| van den Berg 2016 | Low | Low | Some concerns | Some concerns | Low | Some concerns |

Risk of bias evaluation of non-randomised studies using the ROBINS-I tool [2]

|  | Confounding | Selection bias | Bias in classification of interventions | Deviations from intended interventions | Bias due to missing data | Bias in measurement of outcomes | Bias in selection of reported result |
| --- | --- | --- | --- | --- | --- | --- | --- |
| Gräsel 2005 | High | Low | Low | No info | Low | Some concerns | Low |
| Everink 2018 | High | Some concerns | Low | No info | High | Some concerns | Low |
| Harris 2010 | High | High | Low | No info | Some concerns | Low | Some concerns |
| Hebel 2014 | High | High | Some concerns | No info | High | Some concerns | Low |

1 Sterne JAC, Savović J, Page MJ, Elbers RG, Blencowe NS, Boutron I et al. RoB 2: a revised tool for assessing risk of bias in randomised trials BMJ 2019;366:l4898

2 Sterne JAC, Hernán MA, Reeves BC, Savović J, Berkman ND, Viswanathan M et al. ROBINS-I: a tool for assessing risk of bias in non-randomised studies of interventions BMJ 2016;355:i4919
