# Supplementary material for: Can in-hospital or post discharge caregiver involvement increase functional performance of older patients? A systematic review
Source: BMC Geriatr. 2020 Sep 22;20:362. doi: 10.1186/s12877-020-01769-4 (PMC7510152; doi:10.1186/s12877-020-01769-4)
Supplement: Supplementary file 3 — Additional file 3. [file 12877_2020_1769_MOESM3_ESM.docx]

| **Appendix 3. Psychological well-being** | | | | | | | | | | | | | |
| --- | --- | --- | --- | --- | --- | --- | --- | --- | --- | --- | --- | --- | --- |
| **Study Measure points** |  | **Caregiver burden** | | | **Depression of caregiver** | | | **Depression of patient** | | | **Others** | | |
| Everink et al. 2018 |  | **SRCB**, mean (SD) | | |  | | |  | | | **CSAL caregiver,** mean (SD) | | |
| T0= admission geriatric rehabilitation  T1= 3 months  T2= 9 months | **IG**  **CG** | T0  5.5 (2.5)  7 (2.8)  p= 0.29 | T1 4.1 (2.4)  5.4 (2.2)  p= 0.05 | T2 3.5 (2.6)  4.4 (2.2)  p= 0.08 |  |  |  |  |  |  | T0 71 (12.1)  70.9 (13.8)  p= 0.99 | T1  73.2 (15.2)  68.2 (14.3)  p= 0.37 | T2  73.2 (8.2)  68.7 (11.3) p=0.16 |
|  |  |  | | |  | | |  | | | **CSAL patient**, mean (SD) | | |
|  | **IG**  **CG** |  |  |  |  |  |  |  |  |  | T0  65.6 (14.8)  66.4 (12.9)  p= 0.77 | T1 70.7 (9.4)  67.9 (14.1)  p= 0.17 | T2 68.9 (16.4)  71.4 (9.2) p= 0.73 |
| Forster et al. 2013 |  | **CBS**, mean (SE) | | | **HADS** Anxiety, mean (SE) | | | **HADS** Anxiety, mean (SE) | | | **EQ-5D caregiver**, mean (SE) | | |
| T0= baseline  T1= 6 months  T2= 12 months | **IG**  **CG** | T0  45.5 (0.83)  45.0 (0.83)  p= 0.66 | T2  44.8 (0.97) 43.8 (0.96) p=0.435 |  | T1  7.0 (0.23) 7.5 (0.23) p= 0.084 | T2  6.9 (0.26) 7.0 (0.26) p= 0.636 |  | T1  6.7 (0.22) 6.6 (0.21) p=0.629 | T2  6.4 (0.23) 6.6 (0.22) p= 0.355 |  | T1  0.78 (0.011) 0.79 (0.011) p=0.358 | T2  0.81 (0.012) 0.79 (0.012) p=0.240 |  |
|  |  |  | | | **HADS** Depression, mean (SE) | | | **HADS** Depression, mean (SE) | | | **EQ-5D, patient,** mean (SE) | | |
|  | **IG**  **CG** |  |  |  | T1  5.2 (0.22) 5.5 (0.22) p=0.308 | T2 5.2 (0.22) 5.2 (0.22) p= 0.889 |  | T1  7.3 (0.22) 7.2 (0.21) p=0.759 | T2  6.9 (0.25) 7.3 (0.25) p=0.191 |  | T1 0.44 (0.017) 0.44 (0.017) p=0.946 | T2  0.49 (0.019) 0.46 (0.018) p=0.252 |  |
| Galvin et al. 2011 |  | **CSI**, mean change (SD) | | |  | | |  | | |  | | |
| T0= baseline  T1= 8 weeks  T2= 3 months | **IG**  **CG** | T2-T1  -1.3 (1)  -0.2 (1.1)  **p= 0.00** | | |  | | |  | | |  | | |
| Gräsel et al. 2005 |  | **BSFC**, mean change (SD) | | | **ZDS**, mean change (SD) | | |  | | | **GSL (caregiver)**, mean change (SD) | | |
| T0= after intervention  T1=4 weeks  T2= 6 months | **IG**  **CG** | T2-T1 -0.2 (8.6)  -0.1 (10.8)  p= 0.98 | | | T2-T1  -5.3 (26.2)  -5.4 (32.9)  p= 0.153 | | |  | | | T2-T1  7.9 (28.1) 11.1 (29.8) p=0.670 | | |
| Kalra/Patel et al. 2004 |  | **CBS, mean** | | | **HADS Anxiety, median (IQR)** | | | **HADS Anxiety, median (IQR)** | | | **EQ VAS, caregiver, median (IQR)** | | |
| T0= baseline  T2= 12 weeks  T4= 52 weeks | **IG**  **CG** | T2  43  51  **p= 0.0001** | T4  32  41  **p= 0.0001** |  | T4  3 (2 - 4) 4 (3 - 6) **p= 0.0001** | | | T4  IG: 3 (2-4)  CG: 4.5 (4-6)  **p= 0.0001** | | | T0  90 (80-95)  85 (80-90) | T2  80 (71-90)  70 (60-80)  **p= 0.0001** | T4  80 (70-90)  70 (60-80)  **p= 0.0001** |
|  |  |  | | | **HADS** Depression, mean (IQR) | | | **HADS** Depression, mean (IQR) | | | **EQ VAS patient**, mean (IQR) | | |
|  | **IG**  **CG** |  | | | T4  2 (1 - 3)  3 (2 - 5)  **p= 0.0001** | | | T4  G: 3 (2-4)  CG: 4 (2-5.5) **p= 0.0001** | | | T0  85 (75-90)  85 (95-95) | T2  60 (42-70)  50 (40-90)  **p= 0.019** | T4  65 (55-80)  60 (41-80)  **p= 0.009** |
| Van den Berg et al. 2016 |  | **CSI**, mean (95% CI) | | | **HADS**, mean (95% CI) | | | **HADS**, mean (95% CI) | | |  | | |
| T0= baseline  T1= 8 weeks  T2= 12 weeks | **IG**  **CG** | T0  3.6 (2.3-4.9) 3.5 (2.5-4.6)  p= 0.91 | T1  4.9 (3.6-6.2)  4.2 (3.1-5.3)  p= 0.46 | T2  4.4 (3.1-5.7)  3.5 (2.4-4.5)  p= 0.27 | T0  10.1(7.9-12.3)  9.9 (8-11.7)  p= 0.89 | T1  8.1 (5.9-10.4)  10.2(8.3-12.1) p= 0.16 | T2  7.7 (5.5-10)  10.6(8.7-12.5)  **p= 0.05** | T0 10.2(7.7-12.7)  10.8(8.9-12.8)  p= 0.68 | T1  (4.4-9.5)  9.3 (7.3-11.2)  p=0.15 | T2  6.4 (3.9-9)  8.1 (6.1-10.1)  p= 0.29 |  | | |
| IG = Intervention Group, CG = Control Group, SRCB = Self Rated Caregiver Burden, CBS = Caregiver Burden Scale, CSI = Caregiver Strain Index, BSFC = Burden Scale for Family Carers, HADS = Hospital Anxiety and Depression Scale, ZDS = Zerssen Depression Scale, CSAL = Cantril’s Self Anchoring Ladder, EQ-5D = European Quality of Life 5 Descriptive, GSL = Giessen Symptom List, EQ VAS = European Quality of Life Visual Analog Scale | | | | | | | | | | | | | |
