# Supplementary material for: Can in-hospital or post discharge caregiver involvement increase functional performance of older patients? A systematic review
Source: BMC Geriatr. 2020 Sep 22;20:362. doi: 10.1186/s12877-020-01769-4 (PMC7510152; doi:10.1186/s12877-020-01769-4)
Supplement: Supplementary file 4 — Additional file 4. [file 12877_2020_1769_MOESM4_ESM.docx]

| **Appendix 4. Healthcare utilization and other outcome measures studied** | | | | | | | | | | | |
| --- | --- | --- | --- | --- | --- | --- | --- | --- | --- | --- | --- |
| **Study**  **Measure points** |  | **Length of stay** | **Stroke hospitalization costs** | | **Total annual health and social care costs** | **Readmission during one year follow-up** | | **Mortality** | | **Discharge location** | |
| Everink et al. 2018 |  |  |  | |  |  | |  | | **Home**, patients (%) | |
| T0= admission geriatric rehabilitation  T1= 3 months  T2= 9 months | IG  CG |  |  | |  |  | |  | | T1  88 (83) 25 (58.1) **p= 0.004** | T2  94 (88.6) 29 (67.4) **p= 0.004** |
| Forster et al. 2013 |  |  | **Costs in £,** mean | |  |  | |  | |  | |
|  | IG  CG |  | 13127 12471 p= 0.38 | |  |  | |  | |  | |
| Gräsel et al. 2005 |  |  |  | |  | **Number of patients**, (%) | |  | |  | |
| T1= 4 weeks  T2= 6 months | IG  CG |  |  | |  | T1  3 (9) 2 (7) **p= 0.044** | T2 9 (28) 8 (28) p= 0.569 |  | |  | |
| Kalra/Patel et al. 2004 |  | **Days,** mean (SD) | **Costs in £,** mean (SD) | | **Costs in £,** mean (SD) | **Number of patients**/mean days | | **Number of patients**/n | | **Institution,** patients/n | |
| T2= 12 weeks  T4= 52 weeks | IG  CG | 30.8 (26.5)  43.2 (33.6) **p= 0.000** | £7189 (6177) £10079 (7851) **p= 0.000** | | £10544 (9278)  £14587 (10844) **p= 0.001** | 11 / 10.9  10 / 12.8 | | T2  9/151 9/149 p= 0.98 | T4 16/151 16/149 p= 0.88 | T2,  4/151 9/149 p= 0.076 | T4,  2/151 6/149 p= 0.071 |
| van den Berg et al. 2016 |  | **Days,** mean (95% CI) | |  |  | **Patients**, mean (95% CI) | |  | |  | |
|  | IG  CG | 12.8 (7.6 - 18) 24.7 (14.9 - 34.5) **p= 0.0326** |  | |  | 0.45 (0.09 - 0.81) 1.06 (0.57 - 1.56) **p= 0.0432** | |  | |  | |
